# Maternal CD4+ Cell Count Decline after Interruption of Antiretroviral Prophylaxis for the Prevention of Mother-to-Child Transmission of HIV

**DOI:** 10.1371/journal.pone.0043750

**Published:** 2012-08-27

**Authors:** Didier Ekouevi, Elaine J. Abrams, Malka Schlesinger, Landon Myer, Nittaya Phanuphak, Rosalind J. Carter, MTCT-Plus Initiative

**Affiliations:** 1 MTCT-Plus Initiative Programme, PACCI, Abidjan, Côte d’Ivoire; 2 Unité INSERM 893, Institut de Santé Publique d’Epidémiologie et de Développement (ISPED), Université Victor Segalen, Bordeaux, France; 3 International Center for AIDS Care and Treatment Programs (ICAP), Mailman School of Public Health, Columbia University, New York, New York, United States of America; 4 College of Physicians and Surgeons, Columbia University, New York, New York, United States of America; 5 Department of Biostatistics, Mailman School of Public Health, Columbia University, New York, New York, United States of America; 6 Centre for Infectious Diseases Epidemiology and Research, School of Public Health and Family Medicine, University of Capetown, Capetown, South Africa; 7 Thai Red Cross AIDS Research Centre, Bangkok, Thailand; Tehran University of Medical Sciences, Iran (Republic of Islamic)

## Abstract

**Background:**

We evaluated maternal CD4+ cell count (CD4+) decline after PMTCT prophylaxis in a multi-country HIV care program.

**Methods:**

Analysis was restricted to antiretroviral therapy (ART)-naive, HIV-infected pregnant women with CD4+ ≥250 cells/mm^3^ at enrollment. Single-dose nevirapine (sd-NVP) or short-course antiretroviral prophylaxis (sc-ARVp) with zidovudine (AZT) or AZT + lamivudine (3TC) was initiated in 11 programs while 2 programs offered triple-drug antiretroviral prophylaxis (tARVp) (AZT+3TC+ NVP or nelfinavir). All regimens were stopped at delivery. CD4+ decline was defined as proportion of women who declined to CD4+ <350 cells/mm^3^ or <200 cells/mm^3^ at 24 months. Weibull regression was used for multivariable analysis.

**Findings:**

A total of 1,393 women with enrollment CD4+ ≥250 cells/mm^3^ initiated tARVp (172; 12%) or sc-ARVp (532; 38%) during pregnancy or received intrapartum sd-NVP (689; 50%). At enrollment, maternal median age was 27 years (interquartile range (IQR) 23–30), median CD4+ was 469 cells/mm^3^ (IQR: 363–613). At 24 months post-delivery, the cumulative probability of CD4+ decline to <200 cells/mm^3^ was 12% (95% CI: 10–14). Among a subgroup of 903 women with CD4+ ≥400 cells at enrollment, the 24 month cumulative probability of decline to CD4+ <350 cells/mm^3^ was 28%; (95% CI: 25–32). Lower antepartum CD4+ was associated with higher probability of CD4+ decline to <350 cells/mm^3^: 46% (CD4+400–499 cells/mm^3^) vs. 19% (CD4+ ≥500 cells/mm^3^). After adjusting for age, enrollment CD4+ and WHO stage, women who received tARVp or sd-NVP were twice as likely to experience CD4+ decline to <350 cells/mm^3^ within 24 months than women receiving sc-ARVp (adjusted hazard ratio: 2.2; 95% CI: 1.5–3.2, p<0.0001).

**Conclusion:**

Decline in CD4+ cell count to ART eligibility thresholds by 24 months postpartum was common among women receiving PMTCT prophylaxis during pregnancy and/or delivery.

## Introduction

In resource-limited countries HIV-infected pregnant women not yet eligible for antiretroviral treatment (ART) typically receive short-course antiretroviral (ARV) prophylaxis [1] for the prevention of mother-to-child transmission. In July 2010, the World Health Organization (WHO) proposed new guidelines for the use of ARV drugs for treating pregnant women and preventing HIV infection in infants. Two options of similar efficacy are now offered for women with a CD4+ cell count (CD4+) >350 cells/mm^3^, the current immunologic eligibility threshold for initiating ART [2]: Option A, zidovudine (AZT) during pregnancy plus single-dose nevirapine (sd-NVP) at delivery and daily NVP prophylaxis to the infant during breast feeding or Option B, triple ARV prophylaxis (tARVp) starting at 14 weeks of gestation until cessation of all breastfeeding [2]. Despite widespread scale-up of these new guidelines, the impact of stopping prolonged maternal tARVp among women with high CD4+ is not well described with inconsistent results across studies [3], [4]. Furthermore, in non-pregnant HIV-infected adults, structured interruption of treatment significantly increases the risk of opportunistic disease or death in patients eligible for ART [5], [6], raising the possibility that tARVp may lead to suboptimal long-term outcomes compared to other forms of prophylaxis.

The objective of this analysis was to describe CD4+ decline after discontinuation of ARV prophylaxis post-delivery among HIV-infected women not eligible for ART in a large, multi-country cohort of HIV-infected pregnant women and to compare whether this decline varied by PMTCT regimen.

## Methods

### Study Design and Setting

The MTCT-Plus Initiative provided support to 13 clinical programs in eight countries in sub-Saharan Africa (Cameroon, Côte d’Ivoire, Kenya, Mozambique, Rwanda, South Africa, Uganda, and Zambia) and in Thailand to implement HIV/AIDS care and treatment to families identified through PMTCT services [7]. Pregnant or recently postpartum women identified as HIV-infected (HIV+) in PMTCT programs were invited to enroll in the MTCT-Plus Initiative which was built upon existing services and provided HIV-infected women, their partners, and their children, holistic family care with unrestricted access to ART for eligible patients [8], [9].

ART eligibility was based on WHO and national guidelines in effect at the time. Prior to January 2005, criteria included CD4+ <200 cells/mm^3^, WHO stage 4, or CD4+ between 200 and 350 cells/mm^3^ and WHO stage 2 or 3 [1]. After January 2005, women with CD4+ between 200 and 350 cells/mm^3^ and stage 2 were no longer considered ART-eligible.

ART-naïve pregnant women who were not eligible for ART, received an ARV prophylaxis regimen, had a CD4+ prior to or within 30 days of delivery ≥250 cells/mm^3^ and had at least one CD4+ after delivery were included in this analysis. Women receiving ART during pregnancy, or who had CD4+ <250 cells/mm^3^ at enrollment or who had no ARV prophylaxis or missing documentation of ARV prophylaxis were excluded.

### Enrollment and Follow-up

The MTCT-Plus Initiative procedures for enrollment and follow-up have been previously described [8]–[10]. In brief, pregnant women received CD4+ cell count at enrollment and maternal clinical and socio-demographic characteristics were recorded. HIV-infected women not eligible for ART were scheduled to attend follow-up visits every three months for the first six months following enrollment then every six months thereafter for clinical examination, WHO staging and CD4+ monitoring.

### Antiretroviral Prophylaxis for PMTCT

MTCT-Plus Initiative sites followed local guidelines for PMTCT prophylactic regimens. Initially, most programs offered a sd-NVP regimen. By 2004, sites in Bangkok, Thailand, and Eldoret, Kenya, offered triple-drug ARV prophylaxis (tARVp) consisting of zidovudine (AZT) + lamivudine (3TC) + NVP or nelfinavir (NFV) prophylaxis during pregnancy. Over time most sites offered short-course (sc-ARVp) multidrug PMTCT regimens (antepartum AZT with or without 3TC + intrapartum sd-NVP). All ARV prophylaxis initiated antepartum or intrapartum was discontinued after delivery, as per WHO guidelines [1] including tARVp in women not eligible for ART.

### Outcomes

The primary outcome, CD4+ decline, was defined as: 1) the 12 and 24 month cumulative probability from delivery (time when ARV prophylaxis was discontinued) to decline in CD4+ to <200 cells/mm^3^ (2006 WHO recommended eligibility criteria for ART initiation) among women with enrollment CD4+ ≥250 cells/mm^3^; and 2) the 12 and 24 month cumulative probability from delivery to decline in CD4+ to <350 cells/mm^3^ (current WHO recommended eligibility criteria for ART initiation) among women with enrollment CD4+ ≥40 cells/mm^3^.

### Statistical Analysis

Group comparisons were conducted using Student’s t-test or non-parametric Kruskal-Wallis test for continuous variables and the Chi square test or Fisher’s exact test for categorical variables. Kaplan-Meier plots were used to estimate the cumulative probability of reaching outcome. Time was measured from the date of delivery and we right-censored women who were lost-to-follow-up (LTF), (>6 months since last scheduled visit), died, withdrew or were without an event at the date of their last clinical visit or March 31, 2008 when data collection ended. In addition, women who started ART after delivery were censored at the date ART was prescribed. The Log-Rank test was used to compare the probability of CD4+ decline to <200 cells/mm^3^ or <350 cells/mm^3^ between the three ARV prophylaxis groups (sd-NVP, sc-ARVp, and tARVp). We used Weibull regression models to identify risk factors for reaching the criteria for ART eligibility at 24 months. The following variables were considered: ARV prophylaxis regimen, age, CD4+ and WHO stage at enrollment. All factors associated with the outcomes at a p-value <0.25 were included in the multivariable analysis. Adjusted hazard ratios (aHR) and their 95% confidence interval (CI) are reported with two-sided p-values. The effect of program site was evaluated using Kaplan-Meier plots and associated Log-Rank tests independently for each ARV prophylaxis regimen. All analyses were performed in intent-to-treat and on-treatment population with SAS software version 9.2 (SAS Institute, Cary, NC, USA).

### Ethics Statement

The conduct of the MTCT-Plus Initiative as a service delivery program with data collection for monitoring and evaluation purposes was approved by Columbia University’s Institutional Review Board and local ethics review boards in each country where the study was implemented.

## Results

### Enrollment Characteristics

**Figure 1:**
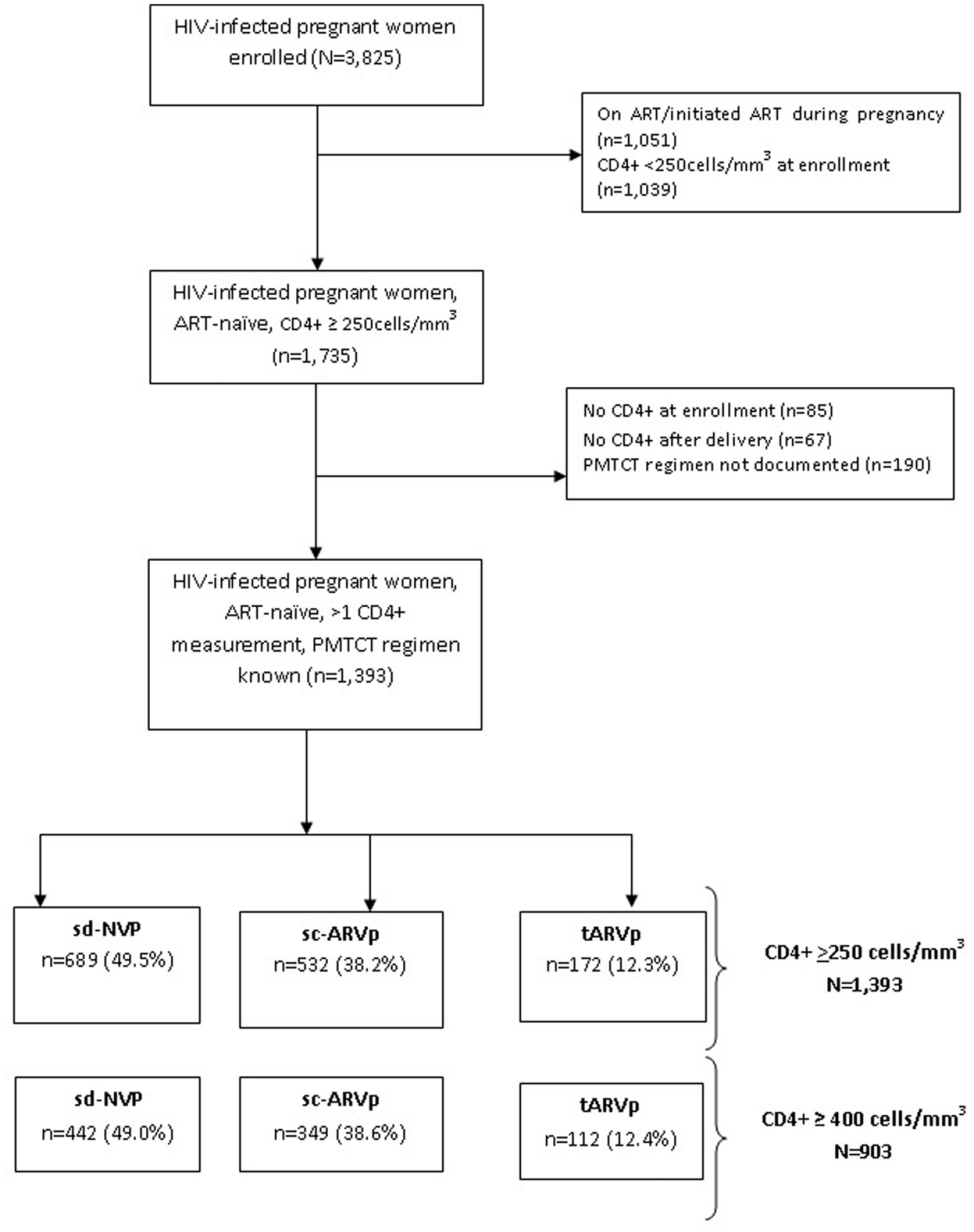
Cohort profile.

Between January 2003 and December 2007, a total of 3,825 HIV-infected pregnant women were enrolled and among them 1,284 initiated ART during pregnancy, 366 had enrollment CD4+ <250 mm^3^ but did not initiate ART and 440 had no CD4+ measurements recorded. After excluding women with only a single CD4+ measure (n = 152) or missing documentation of PMTCT regimen (n = 190), 1,393 women met inclusion criteria for this analysis with enrollment CD4+ ≥250 cells/mm^3^. Among these women a subset (n = 903) also met criteria of CD4+ ≥400 cells/mm^3^ (Figure 1).

Among 1,393 pregnant women who had CD4+ ≥250 cells/mm^3^ at enrollment, 689 (49.5%) received sd-NVP, 532 (38.2%) received sc-ARVp, and 172 (12.3%) women from two programs (Eldoret, Kenya and Thailand) received tARVp. At enrollment, the median age was 27 years (interquartile range (IQR): 23–30), median CD4+ was 469 cells/mm^3^ (IQR: 361–613) and 80.2% of the women were categorized as WHO clinical stage 1 (Table 1). Median CD4+ at enrollment was similar across the three regimen groups (p = 0.36). Women initiating tARVp were slightly older (p = 0.02), more educated (p = 0.001), more likely to be WHO stage 1 (p<0.001), and enrolled in 2004–2005 (p<0.001) compared to women receiving sd-NVP.

**Table 1:** Socio-demographic and clinical characteristics of HIV-infected women with CD4+ cell count ≥250 cells/mm^3^ at enrollment and who initiated PMTCT prophylactic regimens.

	Total	sd-NVP	sc-ARVp	tARVp	p-value
Number	1393	689 (49.5)	532 (38.2)	172 (12.3)	
Age (years)*, n (%)					
Median (IQR)	27 (23–30)	26 (23–30)	27 (24–31)	27 (23–30)	**0.02**
<25	558 (35.3)	264 (38.4)	172 (32.3)	54 (31.4)	0.10
**Education (years), n (%)**					
Median (IQR)	9 (7–12)	8 (7–11)	9 (6–12)	9 (8–12)	**0.001**
12+	363 (22.9)	144 (20.9)	125 (23.5)	39 (20.5)	**<0.001**
Unknown	180 (11.4)	42 (6.1)	107 (20.1)	12 (6.7)	
**CD4+ (cell/mm^3^), n (%)**					
Median (IQR)	469 (361–613)	482 (362–639)	463.5 (363–597)	449 (367–577)	0.36
250–349	353 (22.3)	155 (22.5)	116 (21.8)	33 (19.2)	0.13
350–500	543 (34.3)	217 (31.5)	191 (35.9)	71 (41.3)	
>500	687 (43.4)	317 (46.0)	225 (42.3)	68 (39.5)	
**WHO clinical stage, n (%)**					
Stage 1	1117 (80.2)	566 (82.1)	381 (71.6)	170 (98.8)	**<0.001**
Stage 2	223(16.0)	92 (13.4)	131 (24.6)	0 (0.0)	
Stage 3	52 (3.7)	30 (4.4)	20 (3.8)	2 (1.2)	
Stage 4	0 (0)	0 (0)	0 (0)	0 (0)	
Unknown	1 (0.1)	1 (0.1)	0 (0.0)	0 (0.0)	
**Country, n (%)**					**<0.001**
Cameroon	276 (19.8)	156 (22.6)	120 (22.6)	0 (0.0)	
Côte d’Ivoire	290 (20.8)	17 (2.5)	273 (51.3)	0 (0.0)	
Kenya	237 (17.0)	162 (23.5)	0 (0.0)	75 (43.6)	
Uganda	119 (8.6)	90 (13.1)	29 (5.5)	0 (0.0)	
Mozambique	37 (2.7)	36 (5.2)	1 (0.2)	0 (0.0)	
Rwanda	59 (4.2)	59 (8.6)	0 (0.0)	0 (0.0)	
South Africa	82 (5.9)	11 (1.6)	71 (13.4)	0 (0.0)	
Thailand	133 (9.6)	0 (0.0)	36 (6.8)	97 (53.4)	
Zambia	160 (11.5)	158 (22.9)	2 (0.4)	0 (0.0)	
**Year of enrollment, n (%)**					**<0.001**
2003	181 (13.0)	114 (16.5)	55 (10.3)	12 (7.0)	
2004	408 (29.3)	223 (32.4)	81 (15.2)	104 (60.5)	
2005	326 (23.4)	197 (28.6)	74 (13.9)	55 (32.0)	
2006	250 (17.9)	122 (17.7)	127 (23.9)	1 (0.5)	
2007	228 (16.4)	33 (4.8)	195 (36.7)	0 (0.0)	

PMTCT, prevention of mother-to-child transmission of HIV; sd-NVP, single-dose nevirapine; sc-ARVp, short-course antiretroviral prophylaxis; tARVp, triple-drug antiretroviral prophylaxis; IQR, interquartile range; CD4+, CD4+ cell count.

### Follow-up of Women with CD4+ ≥250 Cells/mm^3^

The median follow-up from enrollment among 1,393 women with CD4+ ≥250****cells/mm^3^ was 27.2 months (IQR: 15.6–41.7) and the median duration of antepartum ARV prophylaxis was 6 weeks (IQR: 4–9), with similar duration for tARVp and sc-ARVp groups (p = 0.052). Overall, 21 (1.5%) women died, and 364 (26.1%) were lost to follow-up over the observation period (2003–2008). The overall loss to follow-up rate was 10.9 per 100 person-years (p–y) with a significantly lower rate among women receiving sc-ARVp (8.0 per 100 py), and similar rates experienced by women receiving sd-NVP and tARVp (12.1 and 12.7 per 100 p–y respectively; p<0.001).

### Incidence of Decline to CD4+ <200 Cells/mm^3^ (WHO 2006 Guidelines)

**Figure 2:**
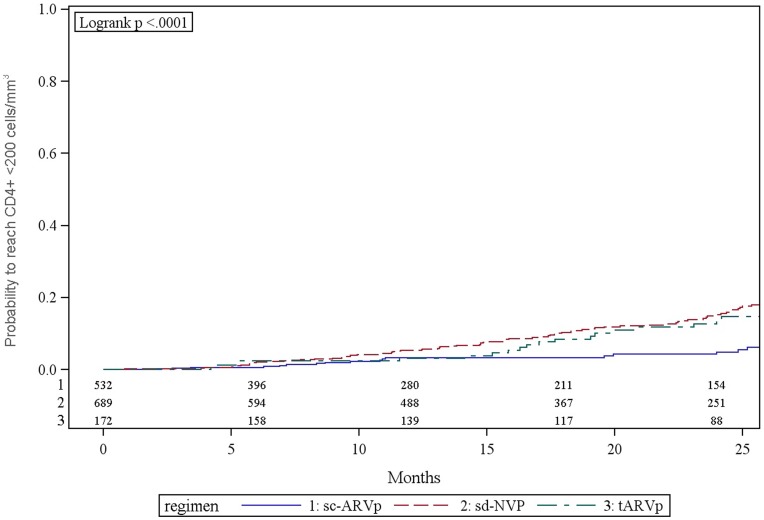
Kaplan-Meier estimate of probability to not reach CD4+ cell count <200 cells/mm^3^ by PMTCT regimens among HIV-infected pregnant women with enrollment CD4+ cell count ≥250 cells/mm^3^.

At 12 and 24 months post-delivery, 4.5% (95% CI: 3.5, 5.8) and 11.6% (95% CI: 9.7, 13.8) of women with enrollment CD4+ cell counts >250 cells/mm^3^ had CD4+ decline to <200 cells/mm^3^ (Figure 2), respectively. Women who received tARVp (13.7%) or sd-NVP (14.9%) had a higher cumulative probability of CD4+ decline to <200 cells/mm^3^ at 24 months compared to those receiving sc-ARVp (4.3%; p<0.0001) (Figure 2). New WHO stage 3 events were experienced by 61 (4.4%) women (6.2% in sd-NVP, 2.6% in sc-ARVp, 2.3% in tARVp groups; p = 0.004) and 5 (0.4%) women reported new WHO stage 4 events (p = 0.44). A total of 218 (15.7%) women initiated ART during follow up: 22.9% in the sd-NVP group, 5.3% in the sc-ARVp and 18.6% in the tARVp; p<0.001.

### Characteristics of Women with CD4+ ≥400cells Cells/mm^3^

Among 903 women with CD4+ ≥400****cells/mm^3^ at enrollment 112 (12.4%) initiated tARVp, 442 (49.0%) sd-NVP, and 349 (38.6%) sc-ARVp. Maternal median age was 26 years (IQR: 23–30), median CD4+ was 568 cells/mm^3^ (IQR: 476–704) and 80.4% were categorized as WHO clinical stage 1 (Table S1). Median CD4+ at enrollment was lower among women initiating tARVp than among women initiating sc-ARVp or sd-NVP: 538 cells/mm^3^ (IQR: 461–642) vs. 557 cells/mm^3^ (IQR: 467–693) and 581 cells/mm^3^ (IQR: 488–734), respectively (p = 0.005). The median duration of antepartum antiretroviral prophylaxis until delivery was 6 weeks (IQR (4–9), with similar duration for tARVp and sc-ARVp groups (p = 0.26).

### Follow-up of Women with CD4+ ≥400 Cells/mm^3^

The median follow-up for 903 women with enrollment CD4+ ≥400 cells/mm^3^ was 26.3 months (IQR, 14.7–41.4): 10 (1.1%) women died, and 262 (29.0%) were reported lost to follow-up. The overall loss to follow-up rate was 12.4 per 100 p–y with a significantly lower rate among women receiving sc-ARVp (8.4 per 100 py), and similar rates experienced by women receiving sd-NVP and tARVp (14.1 and 14.4 per 100 py respectively; p<0.001).

### Incidence of Decline to CD4+ <350 Cells/mm^3^ (WHO 2010 Guidelines)

**Figure 3:**
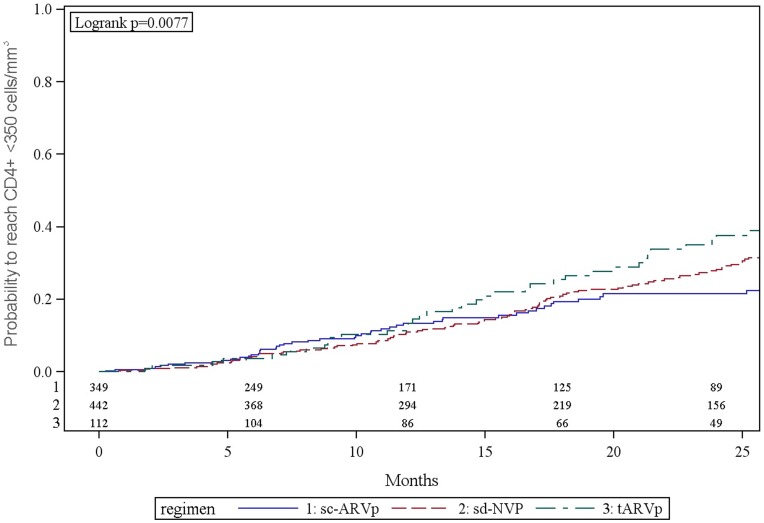
Kaplan-Meier estimate of probability to not reach CD4+ cell count <350 cells/mm^3^ by PMTCT regimens among HIV-infected pregnant women with enrollment CD4+ cell count ≥400 cells/mm^3^.

**Figure 4:**
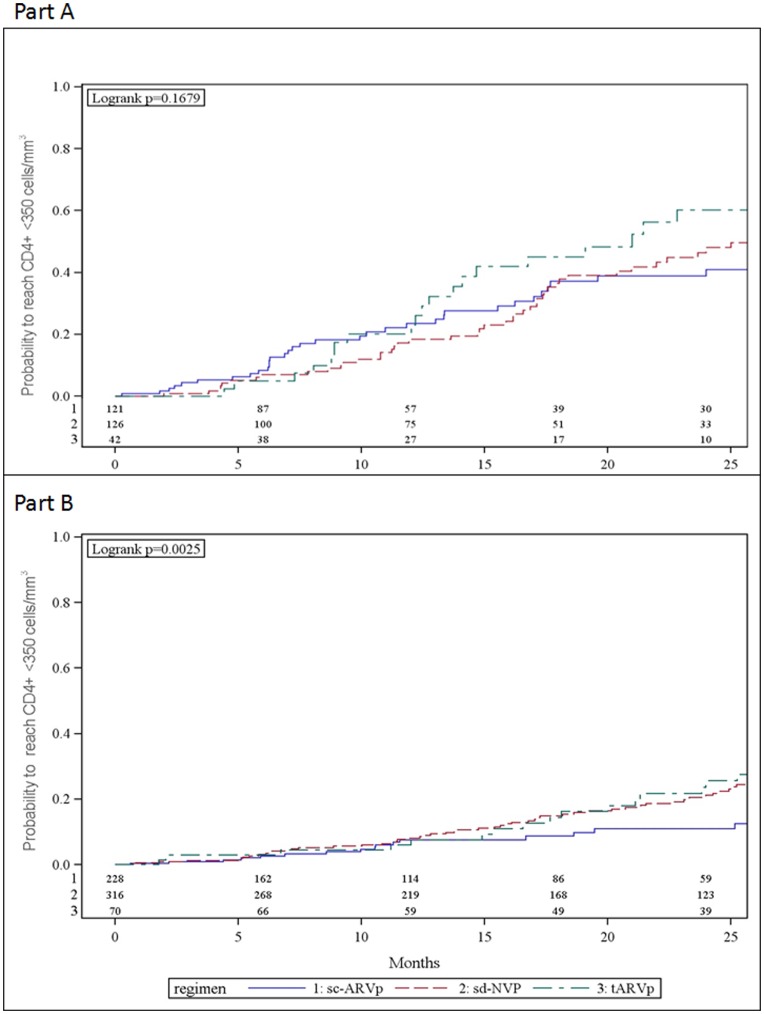
Kaplan-Meier estimate of probability to not reach CD4+ cell count <350 cells/mm^3^ by PMTCT regimens among HIV-infected pregnant women. Part (A) enrollment CD4+ cell count between 400–499 cells/mm^3^ Part (B) enrollment CD4+ cell count ≥500 cells/mm^3^.

Among women who initiated ARV prophylaxis with CD4+ ≥400 cells/mm^3^, the cumulative 12 and 24 month probabilities to reach CD4+ <350 cells/mm^3^ were 11.9% (95% CI: 9.8, 14.3) and 27.5% (95% CI: 24.6, 31.6), respectively (Figure 3). A higher proportion of women whose enrollment CD4+ was 400–499 had CD4+ decline to <350 cells/mm^3^ compared to women with higher enrollment CD4+ (Figure 4A and 4B). At 24 months these probabilities were 46.3% (95% CI: 41.2, 54.8) among women with CD4+400–499 cells/mm^3^ and 18.5% (95% CI: 14.9, 22.3) among women with CD4+ ≥500 cells/mm^3^. Overall 28 (4.4%) experienced new WHO stage 3 events with no differences by PMTCT prophylaxis group (p = 0.44). No new WHO stage 4 events occurred.

Women who initiated tARVp had a higher probability of decline to CD4+ <350 cells/mm^3^ by 24 months compared to women receiving other PMTCT regimens: 36.3% (95% CI: 27.4, 47.2) vs. 21.5% (95% CI: 16.4, 27.9) for women who initiated sc-ARVp and 27.8% (95% CI: 23.0, 33.2) for women who initiated sd-NVP (p = .0077) (Figure 3). Among women who initiated tARVp, 60.3% (95% CI: 43.5, 77.5) with baseline CD4+400–499 cells/mm^3^ had CD4+ decline to <350 cells/mm^3^ by 24 months after delivery compared to 23.6% (95% CI: 14.6, 36.7) with baseline CD4+ ≥500 cells/mm^3^ (Figure 4A and 4B).

Additional analyses examined whether there were significant site differences in the proportion of women reaching CD4 decline <350 cells/mm^3^ endpoint (Table S2). The proportion of women reaching <350 cells/mm^3^ at 24 months post enrollment was similar among women receiving sc-ARVp in Thailand compared to women receiving the same regimen in other sites. Similarly, the proportion of women reaching <350 cells/mm^3^ at 24 months post enrollment was similar among women receiving sd-NVP in Eldoret compared to other sites.

### Multivariate Analysis

Factors associated with CD4+ decline to either <200 cells/mm^3^ or <350 cells/mm^3^ were similar. In adjusted models, ARV prophylaxis regimen, age and enrollment CD4+ were significantly and independently associated with CD4+ decline to <200 cells/mm^3^ and CD4+ decline to <350 cells/mm^3^ (Table 2). Higher CD4+ cell count at enrollment was associated with a reduced probability of immunologic decline to endpoint: for each increase of 100 cells/mm^3^, the probability of CD4+ decline was reduced by 40% for CD4+<200 cells/mm^3^ endpoint, and 30% for decline to CD4+<350 cells/mm^3^. Both women receiving tARVp or sd-NVP were twice as likely to experience CD4+ decline compared to women receiving sc-ARVp: (aHR: 2.3 (95% CI: 1.3,4.2) and aHR: 3.1 (2.0, 4.8) respectively for CD4<200 cells/mm^3^ threshold and aHR: 2.2 (95% CI: 1.5,3.2) and aHR 1.7 (1.2, 2.3) for CD4<350 cells/mm^3^ threshold.

**Table 2:** PMTCT regimens and clinical characteristics associated with progression to CD4+ cell count <200 cells/mm^3^ or CD4+ cell count <350 cells/mm^3^.

	CD4+ <200 cells/mm^3^				CD4+ <350 cells/mm^3^			
	HR (95%CI)	p-value	aHR (95% CI)	p-value	HR (95% CI)	p-value	aHR (95% CI)	p-value
**PMTCT regimens**								
sd-NVP	2.5 (1.7–3.9)	**<.00001**	3.1 (2.0–4.8)	**<0.001**	1.3 (1.0–1.8)	**0.05**	1.7 (1.2–2.3)	**0.001**
sc-ARVp	1.0	–	1.0	–	1.0	–	1.0	–
tARVp	1.9 (1.1–3.4)	**0.02**	2.3 (1.3–4.1)	**0.003**	1.8 (1.2–2.7)	**0.002**	2.2 (1.5–3.2)	**<0.001**
**Age (years)**								
<25	1.0	–	1.0	–	1.0	–	1.0	–
25–30	1.1 (0.7–1.5)	0.73	0.9 (0.6–1.3)	0.49	1.5 (1.1–1.9)	**0.008**	1.4 (1.1–1.9)	**0.02**
31–35	1.8 (1.2–2.6)	**0.006**	1.4 (0.9–2.1)	0.17	1.7 (1.2–2.5)	**0.003**	1.6 (1.1–2.3)	**0.02**
36–40	1.1 (0.6–2.1)	0.68	1.2 (0.6–2.3)	0.54	1.4 (0.9–2.2)	0.17	1.3 (0.8–2.1)	0.37
**CD4+ at enrollment**								
For each increase of 100 cells/mm^3^	0.6 (0.5–0.7)	**<0.0001**	0.6 (0.5–0.7)	**<0.001**	0.7 (0.6–0.8)	**<0.001**	0.7 (0.6–0.8)	**<0.001**
**WHO stage at enrollment**								
Stage 1	1.0	–	1.0	–	1.0	–	1.0	–
Stage 2	1.1 (0.7–1.6)	0.65	1.4 (0.9–2.2)	0.12	1.2 (0.8–1.6)	0.34	1.3 (1.0–1.9)	0.09
Stage 3	0.4 (0.1–1.4)	0.15	0.7 (0.2–2.3)	0.60	1.5 (0.9–2.5)	0.16	1.6 (0.9–2.9)	0.09

PMTCT, prevention of mother-to-child transmission of HIV; CD4+, CD4+ cell count; HR, hazard ratio; 95% CI, 95% confidence interval; aHR, adjusted hazard ratio; sd-NVP, single-dose nevirapine; sc-ARVp, short-course antiretroviral prophylaxis; tARVp, triple-drug antiretroviral prophylaxis.

## Discussion

We examined CD4+ decline among women not yet eligible for ART who received ARV prophylaxis during pregnancy through delivery in the MTCT-Plus Initiative, a multicountry HIV care and treatment program. Within the first 24 months postpartum, 11.6% of women had a CD4+ decline to <200 cells/mm^3^. This figure more than doubled to 27.5% of women with CD4+ decline meeting current ART eligibility thresholds (<350 cells/mm^3^). The finding that one in four HIV-infected women who received ARV prophylaxis during pregnancy/delivery become eligible for ART within 24 months of delivery has profound implications for efforts to eliminate perinatal transmission and improve maternal health [11]. The large number of women who experienced CD4+ decline to ART eligibility thresholds and the speed of this decline underscores the urgent need to engage, and retain HIV-infected women identified during pregnancy in ongoing HIV care and treatment with post-partum CD4+ monitoring a critical component of clinical care. In addition, lower enrollment CD4+ cell count (<500 cells/mm^3^) was associated with a higher risk of CD4+ decline, suggesting that these women may benefit from initiation of therapeutic ART during pregnancy rather than prophylaxis.

Several studies have examined CD4+ decline following PMTCT prophylaxis but findings have been inconsistent and difficult to compare due to varying lengths of follow-up, variety of ARV prophylactic regimens and differences in health status at the time of initiating ARV prophylaxis. Similar to our findings, 20% of 75 HIV+ pregnant women in a Brazilian cohort with similar immunologic status (median CD4+ 573 cells/mm^3^) who initiated tARVp had CD4+ decline to <300 cells/mm^3^ within 24 months after discontinuing prophylaxis [12]. Comparisons of CD4+ decline among women who either discontinued tARVp at delivery or continued have yielded conflicting results: over a short follow-up period of 6 months postpartum, women who discontinued tARVp had a higher rate of decline compared to women who continued ART regimens in a Brazilian cohort [13]. In contrast, Watts and colleagues in the US found no difference in the rate of decline in CD4+ over a longer follow-up period, 12 months postpartum, among women who discontinued prophylaxis compared to those who continued regimens, even after stratifying by prophylaxis regimen (scARVp or AZT monotherapy). Neither study reported CD4+ decline using ART-eligibility end points and thus cannot be directly compared to our results. However, data from AIDS Clinical Trials Group 5170 suggest that CD4+ decline following ARV discontinuation is relatively slow with postpartum women losing an average of 12 CD4+ cells per month after stopping tARVp [4].

This analysis noted that CD4+ during pregnancy was independently associated with CD4+ decline. Women with lower CD4+ at enrollment were more likely to have CD4+ decline to ART-eligibility thresholds. Several recent follow-up studies of disease progression among healthy participants in randomized PMTCT clinical trials in Sub-Saharan Africa have similarly found that CD4+ cell count <500 during pregnancy was associated with a significantly higher risk of decline to CD4<350 threshold within 12–18 months after cessation of ARV prophylaxis [3], [14]. The Kesho Bora trial evaluated CD4+ decline after discontinuation of tARVp or sc-ARVp in women with pregnancy CD4+ between 200 and 500 cells/mm^3^. Consistent with our results, CD4+ at time of antiretroviral prophylaxis initiation was independently and significantly associated with overall disease progression [3].

Among women with pregnancy CD4+ <500, 20% had a CD4+ decline to <350 threshold within 12 months of delivery, and almost half of this group met ART threshold by 24 months postpartum. The rapid disease progression in women with lower CD4+ observed in the MTCT-Plus Initiative and HPTN 046 [14] cohorts provides additional evidence of potential benefit of initiating lifelong ART among pregnant women at higher CD4+ than currently recommended [1]. Several countries in Sub-Saharan Africa are considering new approaches to PMTCT including initiating all pregnant women on lifelong ART to optimize maternal health, prevent infant transmissions, and reduce sexual transmission within discordant partnerships [15], [16]. Initiating ART at higher CD4 threshold for pregnant women would also avoid the potential increased risk of opportunistic diseases or death noted in structured treatment interruption among adults [5], [6]. Although discontinuation of ARV prophylaxis after delivery is not equivalent to structured treatment interruption [5], [6], in sub-Saharan Africa where the incidence of pregnancy among HIV-infected women is relatively high (7.8/100 women-years) and the rate of re-initiation of ARV prophylaxis is likely to be high as well, the clinical implications may be similar [8]. In our study, it was reassuring that we observed relatively little clinical disease progression during the postpartum observation period.

PMTCT regimen was independently associated with CD4+ decline with both triple ARV prophylaxis and sd-NVP regimens associated with two-fold increase in CD4+ decline compared to short-course PMTCT after adjusting for pregnancy CD4+ and WHO clinical stage, maternal age and site. One potential explanation for this finding was differential loss to follow-up (LTF) among the three regimen groups. Although the sd-NVP and tARVp regimen groups had higher LTF rates compared to the sc-ARVp group, we found no differences in CD4+ or WHO staging at enrollment between women lost to follow-up by 24 months and women who remained in follow-up and did not experience decline to CD4+ <350 decline either in the total sample, or within regimen groups. Therefore, we believe that differential losses to follow-up among healthier women is not the likely explanation for our findings. It should be noted that the overall LTF rate (26%) in this cohort of healthy pregnant women after more than 24 months of follow-up is substantially lower than the median LTF rate (55%) reported from other pre-ART adult cohorts in Sub-Saharan Africa [17].

The mechanism through which tARVp interruption may be associated with more rapid decline of CD4+ than other PMTCT regimens are not well understood. In pregnant women, a rebound of plasma HIV-1 RNA viral load to pre-treatment levels after ARV prophylaxis interruption has been reported among women who received sc-ARVp with antepartum AZT and sd-NVP [18], [19]. In the context of tARVp, a change in plasma HIV-1 RNA viral load after tARVp discontinuation could be occurring at a higher magnitude than those after sc-ARVp discontinuation. In addition, a transient overshoot of HIV viremia after discontinuation of ART was reported and resembles the viraemic burst during primary HIV infection [20]. In adults, after an interruption of ART, individuals lost on average 20 cells/mm^3^ per week in the first 8 weeks with a subsequent decrease of 2.0 cells/mm^3^ per week [21]. We speculate that a similar rebound viremia may also explain CD4+ decline among women receiving intrapartum sd-NVP, which may be attenuated by several weeks of zidovudine among women receiving sc-PMTCT. Nonetheless, these findings of different outcomes by ARV prophylaxis regimen will need to be confirmed in other studies.

Strengths of this study include its relatively large sample size and long follow-up period, (median 27 months post-delivery). In addition, as the duration of ARV prophylaxis was relatively short, the CD4+ at enrollment likely reflects the CD4+ at delivery [22] when ARV is discontinued therefore allowing a comparison between the three groups. Unlike other evaluations based on clinical trial cohorts, we present more generalizable “field efficacy” from an HIV treatment program, thus providing important information on the rate of CD4+ decline across a varied population of women. Other strengths were that all women were prospectively followed using a standardized data collection form capturing laboratory and clinical data.

The primary limitation of our study is that the variable of interest (ARV prophylaxis regimens) was not randomized leaving open the possibility that ARV regimen group or other characteristics were not strictly comparable. Although the CD4+ at enrollment was statistically different in the three prophylaxis groups, these differences were not large or clinically meaningful. The fact that tARVp was initiated only in one site in Kenya and in Thailand among women not eligible for ART is another limitation. The decision at both of these sites to use tARVp may represent differences in drug access as well as other treatment approaches that we cannot address in this analysis. In general, we observed a consistent rate of CD4+ decline across programs sites although some sites had higher rates of sc-ARVp or sd-NVP use depending on country guidelines. Another limitation is that women in the MTCT-Plus Initiative only received prophylaxis during pregnancy, as per WHO guidelines in place at the time of the program. Therefore, tARVp was administered for a much shorter duration than currently recommended through the duration of breastfeeding [1], [2]. Also, important laboratory measures of HIV disease progression such as viral load monitoring were not available for this cohort.

In summary, our study showed a substantial proportion of women receiving ARV prophylaxis during pregnancy experienced CD4+ decline to ART-eligibility endpoints within 24 months postpartum. Baseline CD4+ significantly affected time for CD4+ progression after stopping ARV prophylaxis for PMTCT. Approximately 46% of women with baseline CD4+ between 400–499 cells/mm^3^ had CD4+ decline <350 cells/mm^3^ by 24 months. Given this rapid rate of immunologic progression, these findings suggest that pregnant women with CD4+ <500 cells/mm^3^ should be considered for lifetime ART initiation. Recent findings from HPTN 052 demonstrated a 96% reduction of HIV transmission among HIV-serodiscordant couples when HIV-infected partners initiated ART when CD4+ <500 cells/mm^3^ providing additional support for continuation of triple-drug ARV regimen for women after delivery, especially those with potential sero-discordant partners [16]. The IMPAACT network PROMISE trial (clinical trial NCT01061151) is poised to address this pressing question and, in the next several years, should be able to inform treatment and prophylaxis recommendations for pregnant women with HIV.

## Supporting Information

Table S1 **Socio-demographic and clinical characteristics of HIV-infected women with CD4+ cell count ≥400 cells/mm^3^ at enrolment and who initiated PMTCT prophylactic regimens.**
(DOC)

Table S2 **Kaplan-Meier estimate of 24 month probability to reach decline to CD4+ cell count of <350 cells/mm^3^ by PMTCT regimens among HIV-infected pregnant women with enrollment CD4+ cell count ≥400 cells/mm^3^.**
(DOC)
