# Supplementary material for: Maternal CD4+ Cell Count Decline after Interruption of Antiretroviral Prophylaxis for the Prevention of Mother-to-Child Transmission of HIV
Source: PLoS One. 2012 Aug 27;7(8):e43750. doi: 10.1371/journal.pone.0043750 (PMC3428298; doi:10.1371/journal.pone.0043750)
Supplement: Table S1 — Socio-demographic and clinical characteristics of HIV-infected women with CD4+ cell count ≥400 cells/mm3 at enrolment and who initiated PMTCT prophylactic regimens. (DOC) [file pone.0043750.s001.doc]

Table S1. Socio-demographic and clinical characteristics of HIV-infected women with CD4+ cell count >400 cells/mm^3^ at enrolment and who initiated PMTCT prophylactic regimens

|  | | | | | | |
| --- | --- | --- | --- | --- | --- | --- |
|  | **Total** | **sd-NVP** | **sc-ARVp** | **tARVp** | **p-value** |  |
| **N** | 903 | 442 (49.0) | 349 (38.6) | 112 (12.4) |  |  |
| **Age (years)*, n (%)** |  |  |  |  |  |  |
| Median (IQR) | 26 (23-30) | 26 (22-30) | 27 (23-31) | 27 (23-30) | **0.020** |  |
| <25 | 335 (37.1) | 183 (41.5) | 114 (32.7) | 38 (33.9) | 0.100 |  |
| **Education (years), n (%)** |  |  |  |  |  |  |
| Median (IQR) | 8 (7-11) | 8 (7-11) | 9 (6-12) | 9 (7.5-12) | **0.002** |  |
| 12+ | 193 (21.4) | 79 (17.9) | 82 (23.5) | 32 (28.6) | **<0.001** |  |
| Unknown | 118 (13.1) | 35 (7.9) | 74 (21.2) | 9 (8.0) |  |  |
|  |  |  |  |  |  |  |
| **CD4+ (cells/mm^3^), n (%)** |  |  |  |  |  |  |
| Median  (IQR) | 568  (476-704) | 581  (488-734) | 557  (467-693) | 538  (461-642) | **0.005** |  |
| 400-500 | 289 (32.0) | 126 (28.5) | 121 (37.4) | 42 (37.5) | **0.040** |  |
| 501-650 | 321 (35.6) | 154 (34.8) | 123 (35.2) | 44 (39.3) |  |  |
| >650 | 293 (32.5) | 162 (36.7) | 105 (30.1) | 26 (23.2) |  |  |
| **WHO clinical stage, n (%)** |  |  |  |  |  |  |
| Stage 1 | 726 (80.4) | 364 (82.4) | 251 (71.9) | 111 (99.1) | **<0.001** |  |
| Stage 2 | 141 (15.6) | 58 (13.1) | 83 (23.8) | 0 |  |  |
| Stage 3 | 35 (3.9) | 19 (4.3) | 15 (4.3) | 1 (0.9) |  |  |
| Stage 4 | 0 (0.0) | 0 (0.0) | 0 (0.0) | 0 (0.0) |  |  |
| Unknown | 1 (0.1) | 1 (0.2) | 0 (0.0) | 0 (0.0) |  |  |
|  |  |  |  |  |  |  |
| **Country, n (%)** |  |  |  |  | **<0.001** |  |
| Cameroon | 184 (20.4) | 98 (22.2) | 86 (24.6) | 0 (0.0) |  |  |
| Côte d’Ivoire | 200 (22.1) | 13 (2.9) | 187 (53.6) | 0 (0.0) |  |  |
| Kenya | 168 (18.6) | 115 (26.0) | 0 (0.0) | 53 (47.3) |  |  |
| Uganda | 76 (8.4) | 53 (12.0) | 23 (6.6) | 0 (0.0) |  |  |
| Mozambique | 25 (2.8) | 25 (5.7) | 0 (0.0) | 0 (0.0) |  |  |
| Rwanda | 43 (4.8) | 43 (9.7) | 0 (0.0) | 0 (0.0) |  |  |
| South Africa | 43 (4.8) | 8 (1.8) | 35 (10.0) | 0 (0.0) |  |  |
| Thailand | 77 (8.5) | 0(0.0) | 18 (5.2) | 59 (52.7) |  |  |
| Zambia | 87 (9.6) | 87 (19.7) | 0 (0.0) | 0 (0.0) |  |  |
|  |  |  |  |  |  |  |
| **Year of enrollment, n (%)** |  |  |  |  | **<0.001** |  |
| 2003 | 111 (12.3) | 72 (16.3) | 31(8.9) | 8 (7.1) |  |  |
| 2004 | 261 (28.9) | 142 (32.1) | 51 (14.6) | 68 (60.7) |  |  |
| 2005 | 215 (23.8) | 126 (28.5) | 54 (15.5) | 35 (31.3) |  |  |
| 2006 | 164 (18.2) | 76 (17.2) | 87 (24.9) | 1 (0.9) |  |  |
| 2007 | 152 (16.8) | 26 (5.9) | 126 (36.1) | 0 (0.0) |  |  |

PMTCT, prevention of mother-to-child transmission of HIV; sd-NVP, single-dose nevirapine; sc-ARVp, short-course antiretroviral prophylaxis; tARVp, triple-drug antiretroviral prophylaxis; IQR, interquartile range; CD4+, CD4+ cell count
