# Supplementary material for: Maternal CD4+ Cell Count Decline after Interruption of Antiretroviral Prophylaxis for the Prevention of Mother-to-Child Transmission of HIV
Source: PLoS One. 2012 Aug 27;7(8):e43750. doi: 10.1371/journal.pone.0043750 (PMC3428298; doi:10.1371/journal.pone.0043750)
Supplement: Table S2 — Kaplan-Meier estimate of 24 month probability to reach decline to CD4+ cell count of <350 cells/mm3 by PMTCT regimens among HIV-infected pregnant women with enrollment CD4+ cell count ≥400 cells/mm3. (DOC) [file pone.0043750.s002.doc]

Table S2. Kaplan-Meier estimate of 24 month probability to reach decline to CD4+ cell count of <350 cells/mm^3^ by PMTCT regimens among HIV-infected pregnant women with enrollment CD4+ cell count >400 cells/mm^3^

|  | **Total n** | **sd-NVP** | **sc-ARVp** | **tARVp** |
| --- | --- | --- | --- | --- |
|  | N=903 | N=442 | N=349 | N=112 |
| **Country** |  |  |  |  |
| Cameroon | 184 | 23.5 | 10.5 |  |
| Côte d’Ivoire | 200 | 46.1 | 16.6 |  |
| Kenya-Kisumu | 103 | 25.2 | 0 |  |
| Kenya-Eldoret | 65 | 50.0 | 0 | 45.3 |
| Uganda | 76 | 47.1 | 39.1 |  |
| Mozambique | 25 | 16.0 | 0 |  |
| Rwanda | 43 | 32.6 | 0 |  |
| South Africa | 43 | 25.0 | 22.9 |  |
| Thailand | 77 | 0 | 22.2 | 38.9 |
| Zambia | 87 | 35.6 | 0 |  |
